# How many mailouts? Could attempts to increase the response rate in the Iraq war cohort study be counterproductive?

**DOI:** 10.1186/1471-2288-7-51

**Published:** 2007-11-28

**Authors:** A Rosemary Tate, Margaret Jones, Lisa Hull, Nicola T Fear, Roberto Rona, Simon Wessely, Matthew Hotopf

**Affiliations:** 1 King's Centre for Military Health, Research, Institute of Psychiatry, King's College London, London, UK; 2 Academic Centre for Defence Mental Health, Institute of Psychiatry, King's College London, London UK

## Abstract

**Background:**

Low response and reporting errors are major concerns for survey epidemiologists. However, while nonresponse is commonly investigated, the effects of misclassification are often ignored, possibly because they are hard to quantify. We investigate both sources of bias in a recent study of the effects of deployment to the 2003 Iraq war on the health of UK military personnel, and attempt to determine whether improving response rates by multiple mailouts was associated with increased misclassification error and hence increased bias in the results.

**Methods:**

Data for 17,162 UK military personnel were used to determine factors related to response and inverse probability weights were used to assess nonresponse bias. The percentages of inconsistent and missing answers to health questions from the 10,234 responders were used as measures of misclassification in a simulation of the 'true' relative risks that would have been observed if misclassification had not been present. Simulated and observed relative risks of multiple physical symptoms and post-traumatic stress disorder (PTSD) were compared across response waves (number of contact attempts).

**Results:**

Age, rank, gender, ethnic group, enlistment type (regular/reservist) and contact address (military or civilian), but not fitness, were significantly related to response. Weighting for nonresponse had little effect on the relative risks. Of the respondents, 88% had responded by wave 2. Missing answers (total 3%) increased significantly (p < 0.001) between waves 1 and 4 from 2.4% to 7.3%, and the percentage with discrepant answers (total 14%) increased from 12.8% to 16.3% (p = 0.007). However, the adjusted relative risks decreased only slightly from 1.24 to 1.22 for multiple physical symptoms and from 1.12 to 1.09 for PTSD, and showed a similar pattern to those simulated.

**Conclusion:**

Bias due to nonresponse appears to be small in this study, and increasing the response rates had little effect on the results. Although misclassification is difficult to assess, the results suggest that bias due to reporting errors could be greater than bias caused by nonresponse. Resources might be better spent on improving and validating the data, rather than on increasing the response rate.

## Background

Poor response is a major source of concern in epidemiological surveys, and much effort is often spent on chasing up initial non-responders [1] with the implicit assumption that a higher response rate is associated with a more representative sample and hence lower bias. However, there is increasing evidence that this assumption may not always be true. Several reports have found little difference in the risk estimates obtained from the first wave of response and later waves [2-5]. In addition, a recent simulation study by Stang et al [6] suggests that if misclassification error increases with the number of contact attempts, or the prevalence of the exposure decreases, then, if misclassification is non-differential (i.e. independent of exposure status) the estimates after each attempt will become successively biased towards the null hypothesis. Their results are consistent with the long-known fact that non-differential independent misclassification error of a dichotomous outcome will always bias a relative risk estimate for a binary exposure towards the null value (i.e. no difference) [7-9].

While there is an extensive literature on evaluating and dealing with the effects of survey nonresponse (e.g. the collection of articles in [10]) misclassification bias is mostly ignored in the survey literature particularly in relation to attempts to increase response. We could find only a few studies which reported the effect of increasing response on the relative risks e.g. [2-4] and none that explicitly examined whether increasing response rates increased the bias. This was surprising, since the proportion of missing information has been found to be greater for late responders [5,11] which suggests that late responders may take less care in answering a questionnaire and hence make more errors.

To help redress this imbalance, we report an empirical evaluation of the effect of nonresponse bias and outcome misclassification on the relative risks of two health outcomes which were obtained from a recent large study of the health of United Kingdom (UK) military personnel deployed to the 2003 Iraq war [12]. In the first part of this study we attempt to assess the effect of nonresponse bias on the results by comparing the known characteristics of responders and non-responders. In the second part we investigate the pattern of misclassification and prevalence of health risk factors in those who responded. We compare relative risks that were observed with those simulated using Stang's algorithm in an attempt to ascertain the effect of reporting errors across successive waves of response, and whether increasing the initial response rate of 43% to 60%, by numerous and diligent attempts at contact could possibly have been counterproductive.

## Methods

### Data and measures used

#### For investigation of nonresponse bias

We examined data on 17,370 personnel who had been sampled for the first wave of data collection of the Iraq war cohort study. All personnel had been employed in the military between January 18th and June 28th 2003: 7,621 (labelled Op TELIC 1) were recorded as having been deployed in Iraq during this period and 9749 (labelled Era). were not recorded as having been deployed on Op TELIC1. Participants were contacted by post, or were asked to complete a questionnaire during military unit visits made by the research team. Up to 5 further attempts were made to recruit initial non-responders. Reservist personnel were over-sampled by a ratio of 2:1. The study received approval from the Ministry of Defence (Navy) personnel research ethics committee and the King's College Hospital local research ethics committee. Full details of the study design, the participants and the questionnaire are described in [12].

129 personnel who appeared to have never received a questionnaire (i.e. all mailings were listed as return to sender, or they had been recorded as absent during a military unit visit) were excluded as were 42 who were recorded as having died during the study and 166 (1%) who refused to take part in the study. Of the remaining 17,162 personnel, 10,256 (60%) were listed as having returned the questionnaire and were labelled 'responders'.

Demographic information, including age, rank, Service and address, for individuals in our sample was provided by the Defence Analytical Services Agency (DASA), who also provided a monthly fitness category for each person, indicating whether or not they were fit for active duty during that month, known in military jargon as "downgrading status". This study is unusual in that we were able to ascertain the health of non-responders for over two years following the start of the study. Fitness data were available for 99% of regulars and for 55% of reservists. For the purpose of this study 'fit' was defined as fit to deploy at all times between May 2003 (end of TELIC 1) and August 2005. Reservists were excluded from all analyses using the fitness data because of the large percentage with missing data. They were, however, included in all other analyses since reservists showed the biggest health differences between TELIC 1 and Era.

#### For investigation of bias across response waves

For this part of the analysis we used data on the response patterns, fitness indicators and replies to health questions of 10,234 survey participants (labelled 'full responders') after excluding 18 responders who completed only the first page of the questionnaire. These respondents had been sent (or believed they had been sent) the incorrect questionnaire, i.e. a questionnaire tailored for the TELIC 1 group when they had not been deployed on TELIC 1. A further 57 responders were re-assigned from the TELIC 1 to Era group and 22 individuals from Era to the TELIC 1 group after establishing that they had been wrongly classified [12].

The paper by Stang et al., on which we based the simulations, considers error in the exposure variable, for example alcohol consumption, and assumes that the outcome, for example liver cancer, is known. Since the exposure (deployment on TELIC 1) is known in the Iraq war study, we are concerned with misclassification of outcome, but the same principles will apply [13]. We consider two health outcomes: multiple physical symptoms (18 or more physical symptoms) and post-traumatic stress disorder (PTSD) defined as having a score of 50 or more on the Post traumatic Check List (PCL), a commonly used measure of PTSD [14] We have defined outcome misclassification as "errors caused by carelessness in completing the questionnaire." Another possibility would have been to define misclassification as under or over-reporting of multiple physical symptoms. However, since the purpose of the Iraq war study was to identify people who perceived that they had a health problem, rather than to identify those that had some quantifiable disease, the first definition seemed more apt for this investigation. We used two measures for assessing the extent of misclassification: 1. the percentage of discrepant answers to a question on health that asked a similar question in a different way: and 2. the percentage of missing answers to PTSD, and other health questions. For the first measure respondents were labelled 'discrepant' if they gave the same (contradictory) answer to the two questions "I'm as healthy as anyone I know" and "I seem to get ill more easily than other people," where the choice of answers were "definitely true", "mostly true", "mostly false" or "definitely false" [15]. For this measure two variables were constructed, 'discrepant 1', excluded any missing values for the two questions, and 'discrepant 2' labelled those with missing values for both questions as discrepant. For the second measure, having missing health data was defined as falling into at least one of the following categories: 1. having at least 4 missing answers to either the PTSD or General Health Questionnaire 12 [16]; 2. not answering either of the two questions described above; 3. not answering a question on general health. The questions on multiple physical symptoms were not included in this measure since participants were only required to respond to this question if they had at least one symptom. Full details on all the questions on health are provided in [12].

As in [6] wave was defined as the number of contacts that were needed before a successful response, after excluding any attempts where the questionnaire was returned to sender, or the person was listed as being not present at a unit visit (e.g. wave 1 respondents are those that responded at first contact). Two measures were used to assess prevalence of the outcomes, those obtained from the questionnaires, and the fitness category for each person. Although previous evidence has shown that the correlation between fitness status and perceived health may be quite weak [17], fitness status will provide some indication of the likely physical and mental health levels of respondents at each wave.

### Analysis

#### Statistical analysis

Statistical analyses were carried out using Stata 9 (Stata Corporation, Texas, USA), using the svy commands and sampling weights to adjust for the oversampling of reservists.

The factors which differed between responders and non-responders were identified using the chi-squared test and a multivariable logistic regression model, based on these factors (including any significant interactions), was used to predict the probability of response. These probabilities were used to construct an inverse probability weight for each responder, which was then multiplied by the sampling weight. Relative risks for the main health outcomes were estimated with and without response weights and compared in order to determine the extent of nonresponse bias.

All relative risks were estimated using Poisson regression [18]. The estimates of relative risks across response waves were adjusted for age, sex, rank, service type, and reservist status but (in contrast to [12]) we excluded any covariates that might be misclassified and hence cause extra bias [13]. The Rao and Scott second order correction was used for Chi squared tests and an extension of the Wilcoxon rank-sum test was used to test for trends. Sample weights were used for all analyses (and reported percentages) except tests for trend and the Spearman correlation. All reported p values are two-sided.

#### Simulations

The equation presented on page 206 of [6] was used 1. to simulate the 'true' (unbiased) relative risks that would have been observed at wave 4 (for all responders) if there had been no misclassification and 2. to simulate the biased 'observed' relative risks for wave 1 – wave 3 that would result from these 'true' relative risks for a range of 'true' prevalence rates. We compared the simulated observed relative risks with those estimated from the data. We used the proportion of discrepant answers and missing data as measures of misclassification (unlike [6] who used hypothesised specificity and sensitivity). Full details of the calculations are provided in the additional material (see Additional file 1). The R programming language was used for all the simulations [19].

## Results

### Comparison of responders with non-responders

The response rate to the survey was 60%. All of the factors we investigated were related to response (Table 1) except fitness status (p = 0.5), with 22.6% of responders and 22.3% of non-responders labelled as being unfit anytime between May 2003 and August 2005.

**Table 1:** Response rates according to demographic and other factors. Response differed significantly for all factors shown ((p < 0.001)

		Total N	Percentage (weighted) who responded
Age (years) at 01/01/05	<25	3,442	50
	25–29	3,347	58
	30–34	3,432	64
	35–39	3,173	67
	40–49	3,137	64
	50–60	631	69
Gender	Male	15,585	60
	Female	1,577	67
Service	Naval Service	2,943	57
	Army	10,936	61
	RAF	3,283	61
Rank	Officer	2,718	70
	Rank	14,444	59
Status	Reservist	2,987	53
	Regular	14,175	61
Deployment	Era	9,627	58
	TELIC 1	7,535	63
Ethnic group*	White	14,142	62
	Non-white	882	56
All addresses military	Yes	12,705	70
	No	4,457	30
Military unit visited	Yes	5,252	68
	No	11,910	57

	Total	17,162	60

*Ethnic group was missing for 2456 personnel.

Weighting to account for these factors (except ethnic group which had 14% missing) had little effect on the relative risks. The relative risk for multiple physical symptoms by deployment status was 1.19 (95% confidence interval: 1.07, 1.34) using sample weights alone and 1.19 (1.06, 1.33) when nonresponse weights were employed. For PTSD, the relative risks were 1.17 (0.96, 1.43) and 1.15 (0.94, 1.42) respectively.

### Investigation of responses

72% of the participants responded at first contact, and 88% had responded after one reminder (wave 2). 11% of individuals were classified as having multiple physical symptoms and 4% were categorized as having PTSD. Those labeled as unfit were two and a half times as likely to have multiple physical symptoms and 3 times as likely to be classified with PTSD. However, the number of symptoms and PTSD score were only weakly correlated with fitness status (with Spearman correlation coefficients of -0.2).

The percentage of full respondents who gave the same answer to the two health questions was 11.8 increasing to 13.2 when those with missing answers to both questions were included. These percentages were the same for mail and unit visit responses. The most common pair of discrepant answers to the two questions: "I get ill more easily than other people" and "I am as healthy as anyone I know" was "mostly false" (6.5%) followed by "definitely false" and "mostly true" (both 2.6%), with only 0.2% answering both questions "definitely true". There were 2.7% with missing answers for at least one of the two questions and 1.7% with both. There were slightly fewer discrepancies in the TELIC 1 cohort; 10.9% TELIC 1 vs. 12.6% Era (p = 0.01). This difference was mainly due to the smaller percentage of TELIC 1 personnel answering "definitely false" to both questions (1.7% vs. 3.3%). These differences held after adjustment for the other only factors found to be related to discrepancies, i.e. lower rank, and Service (the Army had the highest percentage). However, the percentage with missing answers to both questions was significantly (p = 0.02) greater for TELIC 1 than Era (2.1% vs. 1.5%).

When the discrepancy variable was recalculated to include those with missing data for both questions the difference between TELIC 1 and Era was reduced to 12.6% versus 13.7% and became less significant (p = 0.11). For the purpose of this study, we shall assume that this measure is non-differential between TELIC 1 and Era.

### Investigation of misclassification bias across response waves

The percentage of people giving discrepant answers to the health questions did not change significantly with number of contact attempts, unless those who had missing data for both of the two questions were included as discrepant, when there was a significant upward trend (Table 2). There was also an upward trend in missing answers to any health question (Table 2).

**Table 2:** Trends in discrepancies, PTSD data, fitness status and health outcomes by response wave (number of times a person was contacted before response)

**Number of contacts**	**1**	**2**	**3**	**4+**	**All**	**p-value**
Full responders (N)	7,384	1,651	758	441	10,234	

**Indicators for misclassification**						
Discrepant 1 (%)	11.8	11.1	13.1	12.3	11.8	0.5
Discrepant 2 (%)	12.8	12.9	15.8	16.3	13.2	0.007
At least 1 health question missing (%)	2.4	4.0	5.6	7.3	3.1	<0.001

**Indicators for outcome prevalence**						
Unfit (%)	22.8	21.8	22.3	21.7	22.4	0.4
PTSD 50+ (%)	3.7	4.7	4.1	3.3	3.9	0.6
Symptoms 18+ (%)	10.8	11.7	11.1	9.8	10.9	0.97

Estimates are weighted to allow for oversampling of reservists. p-values are for trend

Those with missing values to either question are excluded from discrepant 1, but included as discrepancies in discrepant 2

Since there was no apparent trend between number of attempts at contact and fitness status, PTSD or multiple physical symptoms (Table 2) we assumed that the true and observed prevalence of both outcomes was constant across wave.

#### Comparison of observed and simulated relative risks across response wave

Table 3 shows the (adjusted) observed cumulative relative risks of the two health outcomes by response wave, showing that these risks are slightly higher at wave 1 than wave 4.

**Table 3:** Cumulative observed relative risks* and 95% confidence intervals for health outcomes over response wave.

**Outcome**	**Number of contacts**							
	1		2		3		4+ (all)**	

Symptoms >17	1.24	1.09, 1.42	1.23	1.09, 1.39	1.21	1.08, 1.36	1.22	1.09, 1.36

PTSD	1.12	0.89, 1.43	1.15	0.93, 1.41	1.13	0.92, 1.38	1.09	0.89, 1.33

N	7384		9035		9793		10234	

Estimates are weighted to allow for oversampling of reservists.

*Adjusted for age, sex, rank, service type, and reservist status.

**These estimates are slightly different from those reported in [12] because we use relative risks rather than odds ratios, and also because we have excluded any confounders that were likely to be misclassified which could cause extra bias.

Since the main aim was to assess the change in relative risk by response wave, and because we needed a non-differential measure, we chose to use the percentage of discrepancies which included missing answers (discrepant 2) at each wave as the hypothesised misclassification rate. This measure had the advantage that it represents the worse case scenario and provides an upper bound for the percentage of true misclassification. The true relative risks that would lead to the observed relative risks at wave 4, i.e. 1.22 for multiple physical symptoms and 1.09 for PTSD if misclassification was 13.6% are shown in Table 4 column 3 for a range of true prevalence rates. This shows that the effect of misclassification decreases with increased true prevalence, so for example, a true prevalence of 8% for multiple physical symptoms would mean that the true relative risk was nearly double that observed, while a true prevalence of 16% would only increase it by 20%.

**Table 4:** Simulated true relative risks (RR's) for multiple physical symptoms and PTSD for a range of hypothesised true prevalence rates. The calculations are based on Stang's algorithm (using an iterative approach to obtain the true RR's).

**Outcome**	**True prevalence (%)**	**True RR**	
**Multiple symptoms:**		**13.22% error**	**6.5% error**
Observed prevalence 10.84%	6	3.05	1.48
Observed RR 1.22	8	2.15	1.41
	10	1.83	1.35
	12	1.65	1.31
	14	1.52	1.28
	16	1.44	1.26

**PTSD**			
Observed prevalence 3.90%	3	N/A	5.5
Observed RR 1.09	4	N/A	1.64
	5	N/A	1.35
	7	N/A	1.18
	9	N/A	1.12
	11	1.90	N/A
	13	1.25	N/A
	15	1.16	N/A

* N/A indicates that the true prevalence for this row is not compatible with the specified error rate

The lowest true prevalence rate for PTSD, compatible with 13.6% misclassification, was 11%. Since it seems unlikely that the true prevalence of PTSD was over three times that observed, we repeated the simulations using a more conservative estimates for misclassification of 6.5%, i.e. half that of discrepant 2 (Table 4 column 4). The prevalence range compatible with this percent was more plausible (3–9%). Even though the difference between the true and observed relative risks is much smaller, the difference is still large when the true prevalence is small, most notably for PTSD at the lowest end of the compatible range (3%) which is associated with a low (and possibly implausible) true positive rate of 0.2%.

The simulated true relative risks shown in column 3 of Table 4 were then used to calculate the cumulative relative risks that would be expected at each wave if the percent of misclassification was the same as discrepant 2 (Table 5). The simulated observed relative risks show a similar pattern of changes as the actual observed relative risks across wave, with the differences across wave becoming less as the true prevalence increases. This same pattern was observed when the percentages of missing data at each wave (which caused the increase in discrepancies by wave) was used to simulate the 'observed' relative risks (data not shown).

**Table 5:** Simulated true and cumulative relative risks (RR's) for TELIC 1/Era that would be observed at each wave if the misclassification rates correspond to the percentage of discrepancies* and the relative risks and the prevalence rates correspond to those observed for multiple physical symptoms and PTSD

**Prevalence (%)**		**True RR**	**Simulated cumulative 'observed' relative risks**			
**Observed**	**True**					
			Wave 1	Wave 2	Wave 3	Wave 4+ (observed)

**Multiple Symptoms**						
10.84	6	3.05	1.26	1.26	1.24	1.22
	8	2.15	1.24	1.24	1.23	1.22
	10	1.83	1.24	1.24	1.23	1.22
	12	1.65	1.24	1.24	1.23	1.22
	14	1.52	1.23	1.23	1.23	1.22
	16	1.44	1.23	1.23	1.22	1.22

**PTSD**						
3.9	11	1.9	1.13	1.13	1.11	1.09
	12	1.40	1.11	1.11	1.10	1.09
	13	1.25	1.10	1.10	1.09	1.09

*Simulations are based on the cumulative percent misclassification of 12.84, 12.85, 13.07, 13.22, i.e. the cumulative percentages of discrepant 2.

## Discussion

We could find no evidence of nonresponse bias in the Iraq war study. In common with most surveys [20], response rate differed significantly according to age, rank (a measure of socio-economic status), gender and ethnic group and also according to cohort enlistment type (regular/reservist), the address type (military or civilian) and whether or not the unit was visited. However, the level of fitness (assessed from downgrading status) was not related to response and adjustment for the factors listed above, using nonresponse weights, made little difference to the results. Although the use of response weights to estimate bias is based on the assumption that the data missing due to nonresponse is ignorable (i.e. that it does not depend on non-measured factors) our findings seem plausible since they are supported by other studies, including that of Klesges et al. [21] who asked US Air Force personnel, who were required to complete a questionnaire on health, whether they would have participated if it had not been compulsory. They found that the risk estimates were similar for those classed as possible responders compared with definite non-responders.

Although difficult to quantify, the percentage of missing answers to health questions suggests that outcome misclassification is at least 3%, and the percentage of responders who gave contradictory answers to two questions asking essentially the same thing, suggests that it could be as high as 14%. A significant upward trend in missing answers suggests that carelessness in answering the questionnaire (our definition of misclassification) increased with response wave. However, simulations based on the percentage of discrepancies and missing answers resulted in only a slight decrease in the relative risks towards the null across response wave. A similar small decrease was observed for the relative risks obtained from the data.

The results of this investigation suggest that, if the assumption of non-differential misclassification and constant prevalence of outcome is correct, the relative risks for health outcomes may be becoming slightly more biased towards the null with each contact. We are aware that the assumption of non-differential error may be unrealistic, since the percentages of both missing answers and discrepancies differ according to deployment status, even though the differences cancelled each other to some extent. This might be due to the fact that personnel deployed on TELIC 1 take slightly more care in answering the questions, but have more doubts on how to complete them. We are also aware that using the discrepant answers to the health questions to assess misclassification was unusual (we could find no other reports that do so). However, the fact that the actual relative risks change little with increasing response does suggest that increasing the response rate using multiple follow-up attempts does not change the bias.

Of greater concern is the extent of misclassification bias. If misclassification is non-differential, the relative risks may be considerably biased towards the null. The simulations demonstrate how a relatively low rate of classification error can cause a large bias in the observed relative risk. For example, if misclassification is 6.5% the simulated true relative risk for PTSD, for a true and observed prevalence of 4%, is 50% larger than that observed. If misclassification is differential, there is still likely to be bias, but it could go in either direction. Estimating the effects of differential misclassification was beyond the scope of this study.

Although there have been various attempts to quantify and correct for misclassification, for example by validating survey answers using data from another source [22,23], such attempts are beset with problems as not only will there be error in the 'gold standard', but it is often difficult to obtain measures that represent exactly the same thing. Indeed a study on a sample similar to that of the Iraq war study [24] found poor correspondence between the questionnaire responses and the reports of the medical officers of the same patients.

## Conclusion

In summary; the results suggest that multiple mailouts were not associated with an increased bias. The estimates changed little over wave, and nearly 90% of participants had responded after one reminder, suggesting that the extra effort to recruit after the second mailing was probably not worthwhile. Although efforts to increase response rates are desirable in order to gain a larger sample and more precise estimates, we suggest that at least equal, if not greater, efforts should be made to assess and to correct for the effects of misclassification bias, for example by using validation data from another source of information, or including items within the questionnaire to be used to check for inconsistent answering.

## Competing interests

The author(s) declare that they have no competing interests.

## Authors' contributions

AR Tate conceived and wrote the paper, and carried out all the analyses. M Jones participated in the conduct of the research, the analysis, and the writing of the paper. L Hull coordinated the study, and was involved in planning the study and writing the paper. NT Fear participated in the planning of the study, made comments on the analysis and contributed to the writing of this paper. R Rona, as a principal investigator, sought funding and participated in the planning, supervision of data collection, and writing the paper. S Wessely, as a principal investigator, sought funding, led the planning of the study and supervision of data collection, and made comments on the analysis and writing of this paper. M Hotopf, as a principal investigator, planned, supervised aspects of data collection, and participated in writing the paper.

## Pre-publication history

The pre-publication history for this paper can be accessed here:



## Supplementary Material

Additional file 1 Supplementary Material: Methods for Simulations. The data provided present the algorithms used for the simulations. Click here for file
