# Supplementary material for: How many mailouts? Could attempts to increase the response rate in the Iraq war cohort study be counterproductive?
Source: BMC Med Res Methodol. 2007 Nov 28;7:51. doi: 10.1186/1471-2288-7-51 (PMC2140265; doi:10.1186/1471-2288-7-51)
Supplement: Additional file 1 — Supplementary Material: Methods for Simulations. The data provided present the algorithms used for the simulations. [file 1471-2288-7-51-S1.pdf]

Supplementary Material: Methods for Simulations

The simulated true and observed relative risks were calculated using this equation:

$$RR_i^{obs} = \frac{\frac{(RR^{true} \times a_i) + c_i}{a_i + c_i}}{\frac{(RR^{true} \times b_i) + d_i}{b_i + d_i}} \tag{1}$$

Where RR_i^{obs} is the observed relative risk, RR^{true} is the true relative risk and a_i is the number of true positives, b_i is the number of false negatives, c_i is the number of false positives and d_i the number of true negatives up to wave i (as shown in Table 1). The $a_i - d_i$'s for each outcome were calculated for a range of true prevalence rates by solving the simultaneous equations:

$$a_i + b_i = true_i \tag{2}$$

$$a_i + c_i = obs_i$$

$$b_i + c_i = misscl_i$$

$$a_i + b_i + c_i + d_i = 100$$

Where true_i, obs_i, and misscl_i were the percentage of true prevalence, observed prevalence and misclassification up to wave i. After calculating these parameters, an iterative application of Equation 1 was used to determine the 'true' relative risk RR^{true} that would lead to the relative risks that were observed for all responders (i.e. at wave 4) for specified prevalence and misclassification rates. A further (direct) application of Equation 1 was then used to calculate the cumulative observed relative risks at each wave which would result from these simulated true relative risks.

Table 1: Notation for observed outcome status of study participants up to wave i

**True status**	**Observed status**	
	% with outcome	% without outcome
% with outcome	a_i	b_i
% without outcome	c_i	d_i
